# The Involvement of Caspases in Neuroinflammation and Neuronal Apoptosis in Chronic Pain and Potential Therapeutic Targets

**DOI:** 10.3389/fphar.2022.898574

**Published:** 2022-05-03

**Authors:** Haoyue Zhang, Nan Li, Ziping Li, Yize Li, Yonghao Yu, Linlin Zhang

**Affiliations:** ^1^ Department of Anesthesiology, Tianjin Medical University General Hospital, Tianjin, China; ^2^ The Graduate School, Tianjin Medical University, Tianjin, China; ^3^ Department of Cardiology, Tianjin Medical University General Hospital, Tianjin, China

**Keywords:** caspase, chronic pain, neuroinflammation, neuronal apoptosis, synaptic plasticity, spinal cord

## Abstract

Chronic pain is a common, complex and unpleasant sensation following nerve injury, tissue trauma, inflammatory diseases, infection and cancer. It affects up to 25% of adults and is increasingly recognized as the leading cause of distress, disability and disease burden globally. Chronic pain is often refractory to most current analgesics, thus emphasizing the requirement for improved therapeutic medications. It is of great importance to elucidate the specific pathogenesis of chronic pain with different etiologies. Recent progress has advanced our understanding in the contribution of neuroinflammation and glial cells (microglia and astrocyte) activation in the plasticity of excitatory nociceptive synapses and the development of chronic pain phenotypes. Oxidative stress-associated neuronal apoptosis is also identified to be a pivotal step for central pain sensitization. The family of cysteine aspartate specific proteases (Caspases) has been well known to be key signaling molecules for inflammation and apoptosis in several neurological conditions. Recent studies have highlighted the unconventional and emerging role of caspases in microgliosis, astrocytes morphogenesis, chemokines release, cytokines secretion and neuronal apoptosis in initiating and maintaining synaptogenesis, synaptic strength and signal transduction in persistent pain hypersensitivity, suggesting the possibility of targeting caspases pathway for prevention and treatment of chronic pain. In this review, we will discuss and summarize the advances in the distinctive properties of caspases family in the pathophysiology of chronic pain, especially in neuropathic pain, inflammatory pain, cancer pain and musculoskeletal pain, with the aim to find the promising therapeutic candidates for the resolution of chronic pain to better manage patients undergoing chronic pain in clinics.

## Introduction

Pain is officially declared as “The Fifth Vital Sign” (Walid et al., 2008). Chronic pain is characterized by pain that sustains or recurs for longer than 3 months (Klein, 2015; Treede et al., 2019). Chronic pain remains to become a major medical issue which affects at least 25% of the general population and imposes a heavy financial burden to patients and healthcare systems worldwide (Gereau et al., 2014; Mills et al., 2019). It frequently presents spontaneous pain, allodynia and hyperalgesia, as a result of nerve injury, cancer, chemotherapy, tissue trauma and inflammation (Ji et al., 2014; Ji et al., 2016; Baral et al., 2019). Patients with chronic pain also often experience insomnia, depression, anxiety and cognitive impairments, which is known to be associated with worsening pain and a serious threat to their quality of life (Moriarty et al., 2011; Tajerian et al., 2014; Tracey et al., 2019). The etiopathogenesis of chronic pain is still debated and, consequently, are the strategies for treating this condition (Ji et al., 2018; Tracey et al., 2019). Chronic pain is identified to be refractory to most analgesics currently (opioids, non-steroidal anti-inflammatory drugs and anticonvulsants) in use (Chou and Huffman, 2007; Tracey et al., 2019), thus emphasizing the urgent need for investigating the specific molecular mechanism that underlies the generation and persistence of chronic pain with different etiology.

Peripheral nociceptive sensitization (trigeminal ganglion and dorsal root ganglion, DRG) and central nociceptive sensitization (spinal cord and brain) mediated changes of neural plasticity in pain neurocircuits contributes to chronic pain phenotypes (Latremoliere and Woolf, 2009; Luo et al., 2014; Bliss et al., 2016; Han et al., 2016; Ji et al., 2018). While acute pain is an essential defensive response involving inflammation, chronic pain that is critically initiated by continuous neuroinflammation can be pathologic and maladaptive (Ji et al., 2016). Neuroinflammation involves glial cells (microglia and astrocyte) activation, chemokines (CCL1, CCL2, CCL7, CXCL1) release and pro-inflammatory mediators (TNF-α, IL-1β, IL-18, BDNF, PGE2) secretion in pain neural circuitry that, subsequently, mediates excitatory neuronal plasticity and synaptic transmission for producing and sustaining chronic inflammatory pain, chronic neuropathic pain, chronic fracture-associated pain, as well as chronic cancer pain (Zhang et al., 2013; Ji et al., 2014; Ni et al., 2019; Qiang and Yu, 2019; Wang et al., 2020b). Accumulating evidence emphasizes that oxidative stress drive neuronal apoptosis and sensitize nociceptors in the pathogenesis of chronic pain, such as chemotherapy-induced peripheral neuropathy (CIPN) and opioid-induced hyperalgesia (OIH) (Zhang et al., 2014; Shu et al., 2015; Grace et al., 2016a; Yousuf et al., 2020; Squillace and Salvemini, 2022). Nevertheless, the involvement of specific molecular signaling in neuroinflammation and neuronal apoptosis remains controversial.

Caspases are a family of conserved aspartate-specific cysteine proteases, which generally exhibits similar structures and presents in the cytoplasm in an inactive form (pro-caspases) (Graham et al., 2011). When an appropriate stimulus is given, caspases will be activated, dimerized and cleaved to form a heterotetramer, the active form of the enzyme (Van Opdenbosch and Lamkanfi, 2019). Activated caspases represent unique catalytic properties and can specifically recognize certain tetrapeptide motifs and cleave an aspartate residue in their substrates, executing programmed cell death (apoptosis) induced by a variety of injuries, including cytokines, chemokines, inflammatory damage and excitotoxicity (Van Opdenbosch and Lamkanfi, 2019). The human caspases family can be subdivided into three functional groups: apoptosis initiator caspases (Caspase-2, 8, 9, and 10), apoptosis effector caspases (Caspase-3, 6, and 7), and inflammatory caspases (Caspase-1, 4, 5, 11, and 12). Initiator caspases elicit the apoptosis signal while the effector caspases carry out the mass proteolysis that leads to apoptosis. Inflammatory caspases do not function in apoptosis but are rather involved in inflammatory signaling and other types of cell death such as pyroptosis (Julien and Wells, 2017).

The caspases have been gradually recognized as a cardinal contributor in neuroinflammatory responses and neuronal apoptosis in a wide variety of neurological and neuropsychiatric disorders, including Alzheimer’s disease (AD), Parkinson’s disease (PD), Huntington’s disease, multiple sclerosis, amyotrophic lateral sclerosis, tauopathies and age-related macular degeneration (Flores et al., 2018; Kirby et al., 2019). Intriguingly, recent progress has advanced our understanding in the unconventional properties of the caspases in mediating the perception of pain. Of note, caspase-1, caspase-3 and caspase-6 are identified as key signaling molecules for nociception induction and persistence by regulating neuroinflammation, neural apoptosis and synaptic plasticity (Berta et al., 2014; Berta et al., 2017a; Gao et al., 2018) in the spinal dorsal horn (Chen et al., 2018; Ji et al., 2018). In this review, we provide a more comprehensive view on the emerging role of caspases in the mechanisms responsible for various pain states. Apart from nerve injury-induced neuropathic pain, we discuss caspases cascades in chronic inflammatory pain, cancer pain, chemotherapy-induced peripheral neuropathy and opioid-induced hyperalgesia. In particular, we summarize the latest basic and clinical advance in this field, and propose the potential therapeutic targets for the resolution of chronic pain in the clinical setting.

## Caspases and Neuropathic Pain

### Caspase-1

Neuropathic pain is primarily triggered by direct nerve trauma in the neurocircuits of peripheral and central somatosensory nervous system. Caspase-1 is the prototypical member of inflammatory caspases involved in cytokine maturation. Dysregulation of inflammasome is strongly associated with the human inflammatory diseases by the enhancement of Caspase-1 activity (Venero et al., 2013). The NOD-like receptor protein 3 (NLRP3) inflammasome are cytosolic multiprotein complexes, which consists of inactive pro-caspase-1 (Liang et al., 2022). When the recruitment of pro-caspase-1 into NLRP3 inflammasome occurs following the exposure to noxious stimuli, pro-caspase-1 will be auto-cleaved to become mature caspase-1 with high bioactivity (Liang et al., 2022). Then, caspase-1 can cleave the pro-IL-1β and pro-IL-18 to generate the activated forms IL-1β and IL-18, further mediating the extracellular secretion of IL-1β and IL-18, which facilitates the transmission of painful information (Rocha et al., 2020; Chen R. et al., 2021). Xu and his colleagues reported that mice with a chronic constriction injury (CCI) of sciatic nerve experience pain-like behaviors followed by the increase of NLRP3 and activated caspase-1 expression in neurons and astrocytes in the superficial dorsal horn of spinal cord, and that pharmacological inhibition of caspase-1 activation attenuates CCI-induced mechanical allodynia and thermal hyperalgesia (Xu et al., 2019). Moreover, Grace et al. (2018) demonstrated that morphine treatment after CCI-induced peripheral nerve injury results in persistent damage associated molecular patterns (DAMP) release, which is critical for formation/activation of spinal caspase-1-dependent NLRP3 inflammasomes, thus causing the extension of CCI induced neuropathic allodynia, whereas inhibition of caspase-1, or IL-1β in the spinal dorsal horn reversed prolonged allodynia. Additionally, morphine induces the phosphorylation of p38, and up-modulates the expressions of Nuclear Factor-κB (NF-κB) subunit P65, toll-like receptor 4 (TLR4) and ionotropic P2X receptors (P2X4 and P2X7) in spinal microglia in CCI rats (Grace et al., 2016b). These alternations are involved in the activation of caspase-1 and the secretion of downstream cytokine IL-1β, which mediates the long-term increase of activity and excitability in nociceptive sensory neurons, and results in the production and persistence of chronic neuropathic pain. In addition to the CCI model, NLRP3 and activated caspase-1 expression were also significantly elevated in spinal glial cells of mice with partial sciatic nerve ligation (pSNL)-induced neuropathic pain (Pan et al., 2018). Meanwhile, the synthesis of caspase-1 in DRG of SNL rats is also increased (Zhang Y. et al., 2015), suggesting that activation of caspase-1 in peripheral nervous system is the pathophysiological basis for pronociceptive hypersensitivity in chronic neuropathic pain. Furthermore, Qian and his colleagues manifest that spinal suppression of caspase-1 activation can effectively reduce the synthesis of cytokines IL-1β and IL-18 and ameliorate mechanical allodynia phenomena in a model of neuropathic pain with spinal cord injury (SCI) (Qian et al., 2017).

In addition to neuropathic pain induced by nerve injury, many antineoplastic agents are capable of eliciting CIPN, which is characterized by mechanical allodynia, a pain evoked by non-nociceptive stimuli such as light touch (Shim et al., 2019). Repetitive injections of intraperitoneal paclitaxel generate and sustain long-lasting CIPN in rats, which is accompanied by NLRP3 over-expression, caspase-1 activation and IL-1β secretion in DRG and sciatic nerve at 3 weeks after paclitaxel application. Furthermore, inhibition of caspase-1 activity reduces the occurrence of CIPN and alleviates the severity of CIPN (Jia et al., 2017). Simultaneously, up-regulations of caspase-1 and NLRP3 in the spinal dorsal horn were verified in oxaliplatin-associated CIPN in rats (Wahlman et al., 2018). The aforementioned findings identified that inhibition of caspase-1-dependent neuroinflammation may offer a novel therapeutic strategy for neuropathic pain control (Table 1).

**TABLE 1:** Caspase-1 and its associated signaling molecules in rodent models of pain.

Pain conditions	Rodent models	Up-Regulation of signaling molecules	References
Neuropathic pain	CCI, C57BL/6 mice	Caspase-1 and NLRP3 in the spinal cord	Xu et al. (2019)
	CCI, C57BL/6 mice	Caspase-1, ASC and NLRP3 in the spinal cord	Tonkin et al. (2018)
	CCI, F344 rats	Caspase-1, DAMP, P2X7R and TLR4 in the spinal cord	Grace et al. (2018)
	CCI, F344 and SD rats	Caspase-1, DAMP, IL-1β, NLRP3, P2X7R and TLR4 in the spinal cord	Grace et al. (2016a)
	CCI, SD rats	Caspase-1, ASC, IL-1β and NALP1 in the spinal cord	Li et al. (2013)
	CCI, SD rats	Caspase-1, ASC, IL-1β, IL-18 and NLRP3 in the spinal cord	Xie et al. (2017)
	CCI, Wistar rats	Caspase-1, MMP-9, IL-1β, IL-6 and IL-18 in the spinal dorsal horn and DRG	Jurga et al. (2017)
	SCI, C57BL/6J mice	Caspase-1, IL-1β and IL-18 in the spinal cord	Qian et al. (2017)
	SNL, C57BL/6J mice	Caspase-1 and NLRP3 in spinal glial cells	Pan et al. (2018)
	SNL, SD rats	Caspase-1 in DRG	Zhang et al. (2015b)
	SNL, SD rats	Cleaved caspase-1, ASC, IL-1β, IL-18, NF-κB, NLRP3 and TNF-α in the spinal cord horn	Wang et al. (2021a)
	CIPN: oxaliplatin, SD rats, C57BL/6 mice	Caspase-1, IL-1β and NLRP3 in the spinal dorsal horn	Wahlman et al. (2018)
	CIPN: oxaliplatin and paclitaxel, C57BL/6 mice	Caspase-1, ASC and NLRP3 in the spinal cord	Tonkin et al. (2018)
	CIPN: oxaliplatin, Swiss mice and C67BL/6 mice	Caspase-1, IL-1β, *GFAP* mRNA and TNF-α in the spinal cord	Agnes et al. (2021)
	CIPN: paclitaxel, SD rats	Caspase-1, IL-1β and NLRP3 in DRG and sciatic nerve	Jia et al. (2017)
Inflammatory pain	Carrageenin injection, C57BL/6 mice	Caspase-1, IL-1β maturation, COX-2 and PGE2 in paw skins	Cunha et al. (2010)
	CFA or ceramide injection, C67BL/6 mice	Caspase-1, IL-1β and NLRP2 in DRG; caspase-1 and NLRP3 in spinal dorsal horn neurons	Matsuoka et al. (2019)
	CFA, SD rats and CB2 receptors KO mice	Caspase-1, ACS, IL-1β and NLRP3 in the skin tissue	Gao et al. (2018)
	Hindpaw incision, C57BL/6 mice	Caspase-1 near the wounds	Liang et al. (2010)
	LPS, Balb/c mice	Caspase-1, ASC, IL-1β and NLRC4 in the brain and spinal cord	Cagli et al. (2021)
	LPS, Wistar rats	Caspase-1, ASC, IL-1β and p-P38 in the spinal cord	Clark et al. (2006)
	MIA knee injection, SD rats	Caspase-1, ASC, IL-1β, IL-18 and NLRP3 in fibroblast-like synoviocytes	Ma et al. (2020)
Cancer pain	Walker 256 cells injection in tibial cavity, SD rats	Caspase-1, ASC and NLRP3 in the spinal cord	Chen et al. (2019)
Postoperative pain	Thoracotomy, SD rats	Caspase-1, IL-1β, IL-6, TLR4, TNF-α and in the spinal dorsal horn	Hu et al. (2020)
	Laparotomy, SD rats	Caspase-1, IL-1β, NF-κB, NLRP3, TLR4 and TNF in the spinal dorsal horn	Grace et al. (2019)

Abbreviations: ASC, apoptosis-associated speck-like protein containing a Caspase activation and recruitment domain; CB2, cannabinoid receptor type 2; CCI, chronic constriction injury; CFA, complete Freund’s adjuvant; CIPN, chemotherapy induced neuropathic pain; CPTP, chronic post-thoracotomy pain; COX-2, cyclooxygenase-2; DAMP, damage associated molecular patterns; DRG, dorsal root ganglion; GFAP, Glial fibrillary acidic protein; IL-1β, interleukin-1β; KO, knockout; LPS, lipopolysaccharide; MIA, monosodium iodoacetate; MMP-9, matrix metalloproteinase-9; NALP1, NACHT leucine-rich-repeat protein 1; NF-κB, nuclear factor-kappa-B; NLRC4, NOD-like receptor C4; NLRP3, NOD-like receptor protein 3; P2X7R, P2X7 receptor; PGE2, Prostaglandin E2; SCI, spinal cord injury; SD rats, Sprague Dawley rat; SNL, sciatic nerve ligation; TLR4, toll-like receptor 4; TNF-α, tumor necrosis factor-alpha.

### Caspase-3

Caspase-3 is known as an executioner caspase in apoptosis because of its potent role in coordinating the destruction of cellular structures such as DNA fragmentation or degradation of cytoskeletal proteins (McIlwain et al., 2015). Caspase-3 is one of the key indicators of apoptosis, and studies in the field of chronic pain with caspase-3 are still in its exploratory stage. Wu et al. (2012) elucidated that sciatic nerve injury induced by the CCI model not only initiates chronic neuropathic pain, but also increases the expression of caspase-3 in the spinal cord and caspase-3-dependent apoptosis of dorsal horn neurons, which is associated with up-regulation of growth associated protein 43 (GAP-43) expression. Behavioral results also indicate that inhibition of caspase-3 activity by both pharmacological therapy with caspase-3 inhibitor Z-DEVD-FMK and caspase-3 knockdown attenuates the thermal hyperalgesia in CCI rats (Wu et al., 2012). Li et al. also found that microRNA-212-3p controls peripheral neuropathic allodynia and sodium voltage-gated channel alpha subunit 3 (Nav 1.3) through inhibiting caspase-3 cleavage and B-cell lymphoma 2-associated X apoptosis regulator (BAX) expression in rats with CCI surgery (Li Y. et al., 2019). EphrinB/EphB signaling, the most important subfamily of receptor tyrosine kinases (RTKs) in humans, is one of pivotal cascades in the spinal pain processing and nociceptive synaptic plasticity (Deng et al., 2017; Peng et al., 2019). Recent evidence discloses that caspase-3 activation in the spinal dorsal horn neurons but not microglia and astrocyte is implicated in EphrinB/EphB signaling dependent neuropathic pain formation in a mouse model of CCI (Yang et al., 2018) (Table 2).

**TABLE 2:** Caspase-3 and its associated signaling molecules in rodent models of pain.

Pain conditions	Rodent models	Up-Regulation of signaling molecules	References
Neuropathic pain	CCI, Kunming mice	Caspase-3, calpain-1 in the neurons of spinal cord	Yang et al. (2018)
	CCI, SD rats	Caspase-3 and GAP-43 in the spinal cord	Wu et al. (2012)
	CCI, SD rats	Caspase-3 and TNF-α in the spinal cord	Hu et al. (2015)
	CCI, SD rats	Cytochrome-C-positive neurons and cleaved caspase-3-positive neurons in the spinal cord	Fu et al. (2017)
	CCI, SD rats	Caspase 3 and HIF-1α in the spinal cord	Mo et al. (2018)
	CPN, C57BL/6 mice	Caspase 3 in the ACC	Wang et al. (2020c)
	SCI, SD rats	Caspase 3, caspase-8, IL-1β and IL-18 in the spinal cord	Turtle et al. (2017)
	SCI, SD rats	Caspase 3, CD68(+), GFAP, iNOS, MDA, NMDAR1, 3-NT, TNF-α in the spinal cord	Lv et al. (2017)
	SCI, SD rats	Caspase-3 mRNA, Bcl-2-associated X protein, COX-2, interleukins, iNOS and TNF-α in the spinal cord	Cui et al. (2021a)
	SCI, Wistar rats	Caspase 3 in the spinal cord	Hajimashhadi et al. (2017)
	SNL, Wistar rats	Caspase-3, ATF-3 and anoctamin-1in DRG	García et al. (2018)
	PSNL, albino mice	Caspase-3, COX-2, IL-1β, IL-6, iNOS, TNF-α in the spinal cord	Khan et al. (2021)
	CIPN: paclitaxel, SD rats	Caspase-3 in DRG	Choi et al. (2013)
	CIPN: paclitaxel, C57BL/6 rats	Caspase 3 and RhoA in DRG	Chine et al. (2019)
	CIPN: paclitaxel, Wistar rats	Caspase 3, NF-kB p65, TNF-α in the sciatic nerve	Al-Massri et al. (2018)
	STZ-induced diabetes, SD rats	Caspase-3, hydroperoxides, lipid peroxides, NOX2 and NOX4 in the sciatic nerve	Ji et al. (2017)
	STZ-induced diabetes, SD rats	Caspase 3, AGE and BAX in the sciatic nerve tissue	Yu et al. (2021)
	STZ-induced diabetes, SD rats	Caspase 3, CX3CL1 in DRG, p38 MAPK in macrophage	Huang et al. (2014)
	STZ-induced diabetes, Wistar rats	Caspase-3 and the Bax/Bcl-2 ratio in the spinal cord	Rasoulian et al. (2019)
inflammatory pain	CFA, C57BL/6 mice	Caspase 3, BAX, NF-KB, NMDAR, TNF-α, P38 phosphorylation in the anterior cingulate cortex	Fan et al. (2018)
Cancer pain	Walker 256 cell intraperitoneal injection, SD rats	Cleaved caspase-3, ATF6, GRP78, p-IRE1 and p-PERK in the spinal cord	He et al. (2019)
	Walker 256 cell injection in tibia cavity, SD rats	Caspase-3, Iba-1, and the mRNA levels of IL-1β, TNF-α and IL-6 in CSF-CN	Chen et al. (2021a)
	MRMT-1 cell injection in tibia cavity, SD rats	Cleaved caspase-3, Bcl-2/BAX ratio and Drp1 in the spinal cord	Li et al. (2019a)
Musculoskeletal pain	Tibial fractures	Caspase-3 and LRRTM1 in the spinal dorsal horn	Zhang et al. (2020)

Abbreviations: ACC, anterior cingulate cortex; BAX, B-cell lymphoma 2-associated X apoptosis regulator; Bcl-2, B-cell lymphoma-2; CCI, chronic constriction injury; CD68(+), CD68-positive cells; CIPN, chemotherapy induced neuropathic pain; COX-2, cyclooxygenases-2; CPN, common peroneal nerve ligation; CSF-CN, cerebrospinal fluid-contacting neurons; CX3CL1, chemokine (C-X3-C motif) ligand 1; DRG, dorsal root ganglion; GAP-43, growth associated protein-43; GFAP, glial fibrillary acidic protein; GRP78, glucose regulatory protein 78; HIF-1α, hypoxia inducible factor-1α; IL-1β, interleukin-1β; iNOS, inducible nitric oxide synthase; LRRTM, leucine-rich repeat transmembrane neuronal protein; MDA, malondialdehyde; NF-κB, nuclear factor-kappa-B; NMDAR, N-methyl-d-aspartate receptor; 3-NT, 3-nitrotyrosine; pSNL, partial sciatic nerve ligation; p-IRE1, phosphorylated inositol-requiring enzyme-1; p38 MAPK, p38 mitogen-activated protein kinase; NOX2, NADPH oxidases 2; p-PERK, phosphorylated protein kinase RNA-like endoplasmic reticulum kinase; SCI, spinal cord injury; SNL, sciatic nerve ligation; STZ, streptozotocin; TNF-α, tumor necrosis factor-α.

Furthermore, spinal caspase-3 cleavage is required for axonal degeneration, mitochondrial dysfunction, oxidative stress and apoptosis in the pathogenesis of CIPN after intraperitoneal vincristine stimulation in mice (Chen et al., 2020). The over-expression of apoptosis-related proteins of BAX, BCL2, and caspase-3 in the sciatic nerve is reported in streptozotocin (STZ)-induced diabetic peripheral neuropathy in rats (Yu et al., 2021). Blocking caspase-3 signaling cascades can reduce spinal neuronal apoptosis and down-regulate nociceptor hyper-responsiveness, which may emerge as a promising strategy for the treatment of neuropathic pain. Conversely, peripheral nerve injury induced by common peroneal nerve (CPN) ligation blocks long-term depression (LTD) induction and caspase-3 expression in the anterior cingulate cortex (ACC). Electrophysiological and behavioral tests found that disrupting the link between caspase-3 and α-amino-3-hydroxy-5-methyl-4-isoxazolepropionic acid (AMPA) receptor in the ACC inhibits LTD expression and induces peripheral pro-nociception phenotypes (Wang YJ. et al., 2020). Restoration of LTD through caspase-3 accumulation in the ACC relieves persistent mechanical allodynia (Wang YJ. et al., 2020), which may be used as a promising therapeutic approach for the management of chronic pain (Table 2). Acetyl-l-carnitine (ALCAR) is a short chain ester of carnitine L-isomer, which is the predominant acyl-carnitine and is involved in the redox reactions to eliminate reactive oxygen species, and finally, can increase acetylcholine levels, thus having neuroprotective action. Di Cesare Mannelli et al. (2007) found the decreased effect of ALCAR on the induction of apoptosis, the release of cytosolic cytochrome C, the activation of caspase-3, and the fragmentation of the genome in CCI rats. And that means ALCAR may be an agent suitable for clinical use in the prevention of nervous tissue cell death after peripheral nerve trauma *via* the inhibition by caspase-3. Di Cesare Mannelli et al. (2013) also found that antioxidants (such as silibinin and α-tocopherol) can ameliorate the symptoms of neuropathy and protect astrocytes from caspase-3-dependent apoptotic signaling activation in oxaliplatin-treated rats.

### Caspase-6

Caspase-6 is also tightly associated with the pathophysiology of neuropathic pain. Peripheral nerve injury after CCI intervention is demonstrated to cause the release of activating transcription factor-3 (ATF3) and caspase-6 from axonal terminal, which then acts on microglia to trigger their activation. After microglial activation, p38 will be phosphorylated to further induce TNF-α release, and brain-derived neuro-trophic factor (BDNF) expression, which inducing central sensitization and supporting the transition from acute pain to chronic pain (Berta et al., 2016). Caspase-6 inhibition significantly reverse the development of mechanical allodynia in a rat model of neuropathic pain following spared nerve injury (SNI) of sciatic nerve. Moreover, caspase-6 deletion attenuated behavioral mechanical allodynia in the paclitaxel-induced CIPN, although the mechanisms that produce neuropathic pain after exposure to chemotherapeutics may be fundamentally different from those operating after nerve injury (Berta et al., 2017b). (Table 3)

**TABLE 3:** Caspase-6 and its associated signaling molecules in rodent models of pain.

Pain conditions	Rodent models	Up-Regulation of signaling molecules	References
Neuropathic pain	SNI, SD rats	Caspase-6 in DRG	Berta et al. (2016)
	CIPN: paclitaxel, C57BL/6 mice, *Casp6* ^–/–^mice	Caspase-6 in DRG	Berta et al. (2016)
Inflammatory pain	Formalin, CFA, C57BL/6 mice, *Casp6* ^–/–^mice	Caspase-6, TNF-α in the spinal cord	Berta et al. (2014)
Musculoskeletal pain	Tibial fracture	Caspase-6, AMPAR-induced current in dorsal horn neurons, GluA1-containing AMPAR trafficking, netrin-1 release, spine density in spinal cord	Cui et al. (2021b)
	C57BL/6 mice		
Opioid-induced hyperalgesia	Remifentanil	Caspase-6, CCL21, CXCR3 in spinal cord	Wang et al. (2020a)
	SD rats		

Abbreviations: AMPAR, alpha-amino-3-hydroxy-5-methyl-4-isoxazolepropionic acid receptor; CCL21=C-C Motif Chemokine Ligand 21; CIPN, chemotherapy induced neuropathic pain; CXCR3, C-X-C Motif Chemokine Receptor 3; DRG, dorsal root ganglion; SNI, spared nerve injury; TNF-α, tumor necrosis factor-α

### Other Caspases

In recent years, in addition to caspase-1, caspase-3 and caspase-6, other caspases have gained great emphasis on neuropathic pain syndromes. Specifically, increase of caspase-9, apoptosis, mitochondrial reactive free oxygen species (fROS), lipid peroxidation, glutathione (GSH), transient receptor potential vanilloid-1 (TRPV1) current density, and calcium concentrations in the DRG and hippocampus was detected in STZ-induced diabetic peripheral mechanical allodynia and thermal hyperalgesia (Düzova et al., 2021). Application of metabotropic glutamate receptor 1 (mGluR1) antagonist can prevent CCI-induced neuropathic pain by reducing the synthesis of caspase-7 in the spinal dorsal horn and inhibiting the process of caspase-7 dependent neuronal apoptosis (Siniscalco et al., 2008). However, whether caspase-9 and caspase-7 inhibitors can provide definitive relief of chronic pain is a scientific question that requires to be addressed.

## Caspases and Inflammatory Pain

### Caspase-1

Inflammatory pain is evoked by inflammation-associated stimuli and is often established in animal models using wound incision or injection of inflammatory chemical irritants, such as complete Freund’s adjuvant (CFA), carrageenan or lipopolysaccharides (LPS) (Pan et al., 2021). Recent literatures have demonstrated that intraplantar administration of complete Freund’s adjuvant (CFA) or ceramide not only down-regulates paw withdrawal mechanical threshold and paw withdrawal thermal latency, but also up-regulates the expression of the NOD-like receptor protein 2 (NLRP2)/caspase-1/IL-1β in small-sized DRG primary sensory neurons and the generation of NLRP3/caspase-1 in spinal dorsal horn neurons (Matsuoka et al., 2019; Hua et al., 2022). Intrathecal injection of a caspase-1 inhibitor Z-YVAD-FMK impairs CFA-induced inflammatory pain hypersensitivity through inhibiting IL-1β secretion in DRG (Matsuoka et al., 2019). Liang et al. also found, in the mouse model of hind paw incision, that caspase-1 activity was significantly increased in peri-incisional tissues. Caspase-1 inhibitor VRTXSD727 significantly reverses mechanical allodynia and thermal hyperalgesia, and reduces the synthesis and release of macrophage inflammatory protein-1α (MIP-1α), granulocyte-colony stimulating factor (G-CSF), Prostaglandin E2 (PGE2), as well as IL-1β around the wound incision (Liang et al., 2010). Additionally, caspase-1 knockout mice exhibit the impairment in the mechanical allodynia induced by intraplantar exposure to carrageenin, TNF-a and exogenous CXCL1, respectively. Meanwhile, caspase-1 deficiency suppresses carrageenin-induced PGE2 production, IL-1β maturation and cyclooxygenase-2 (COX-2) accumulation (Cunha et al., 2010). These detailed evidences strongly suggest the importance of caspase-1-dependent inflammatory cascades in the pathophysiology of inflammatory hyper-nociception (Table 1).

### Caspase-3

Caspase-3 plays an important role in inflammatory pain. N-methyl-d-aspartate (NMDA) receptor, an ionotropic glutaminergic receptor, consists of the primary GluN1 subunit and one or more GluN2A-D modulatory subunits (Zhang et al., 2014). Activation of NMDA receptor is a leading determinant in the hyper-excitability of nociceptive neurons and central synaptic plasticity underlying pain-associated syndromes (Chen et al., 2016; Xu et al., 2020). The excitotoxicity of NMDA receptor is GluN2B dependent (Zhang et al., 2021). Notably, CFA injection into hind paw can not only aggravate neuronal apoptosis but also increase the expressions of Bax, caspase-3 and GluN2B-containing NMDA receptors in the ACC. Inhibiting caspase-3-dependent cascades protects neuronal survival, reduces GluN2B-containing NMDA receptor electrophysiological function and attenuates chronic inflammation-induced mechanical allodynia and thermal hyperalgesia (Fan et al., 2018) (Table 2).

### Caspase-6

In 2014, a preclinical study by Berta et al. showed that caspase-6 is specifically expressed in C-fiber axonal terminals in the superficial spinal cord dorsal horn, and co-localizes with calcitonin-gene-related peptide (CGRP), suggesting the transportation of caspase-6 in peptidergic primary afferents to spinal central terminals, which sustaining nociception-related synaptic potency. Moreover, injections of formalin or bradykinin into the plantar induce the cleavage and activation of caspase-6 in nociceptive neurons of spinal dorsal horn. Spinal application of specific caspase-6 inhibitor Z-VEID-FMK or caspase-6 neutralizing antibody or delivery of caspase-6 siRNA around the sciatic nerve can effectively relieve the inflammatory pain induced by formalin intervention. Similarly, caspase-6 gene knockout reduces bradykinin-induced spontaneous pain, CFA-induced mechanical allodynia, and carrageenan-induced heat hyperalgesia, respectively. In addition, spinal exposure to recombinant caspase-6 not only facilitates microgliosis and microglial activation to result in TNF-α secretion, but also increases glutamate release from primary afferent terminals to enhance excitatory postsynaptic currents (Berta et al., 2014). These detailed results identify that caspase-6 activation may be a predominant feature of neuroinflammation and neuron-microglia interaction, as well as a key driver of synaptic plasticity and central sensitization, thereby mediating persistent inflammatory pain. Thus, targeting the caspase-6/TNF-α cascades may offer a novel choice for treating inflammatory pain states by microglial and synaptic modulation.

### Other Caspases

In addition, recent report recapitulates the elevated concentration of caspase-11, NOD-like receptor C4 (NLRC4), ASC, and IL-1β in the brain and spinal cord of mice with lipopolysaccharide (LPS)-induced inflammatory heat hyperalgesia (Cagli et al., 2021), suggesting the implication of caspase-11-dependent NLRC4 inflammasome in pain perception.

## Caspases and Cancer Pain

### Caspase-1

Cancer pain is also an important category of chronic pain and have the distinctive characteristic of both neuropathic pain and inflammatory pain processing (Wang K. et al., 2020; Wang K. et al., 2021). Mounting evidence reveal that the expression of NLRP3 inflammasome, including NLRP3, apoptosis-associated speck-like protein containing a caspase activation and recruitment domain (ASC), and caspase-1, were significantly increased in a time-dependent manner in bone cancer pain (Chen et al., 2019). Behavioral tests confirmed that both single and repetitive treatment with NLRP3 inhibitor MCC950 markedly attenuated cancer pain behaviors (Chen et al., 2019), suggesting that the activation of NLRP3/ASC/Caspase-1 signaling cascades is an essential step for the initiation and maintenance of central pain sensitization following bone cancer (Table 1).

### Caspase-3

Bioactivity of endoplasmic reticulum (ER) is vital for life yet toxic if dysfunction of ER occurs. Especially, ER stress plays a positive role in acute pain perception and chronic pain sensitization (Zhang E. et al., 2015; Inceoglu et al., 2015). Bone cancer pain not only induces bone destruction and unbearable mechanical allodynia, but also up-regulates the spinal expression of ER stress markers, including glucose-regulated protein 78 (GRP78), activating transcription factor-6 (ATF6), phosphorylated protein kinase RNA-like endoplasmic reticulum kinase (p-PERK), phosphorylated inositol-requiring enzyme-1 (p-IRE1) and cleaved caspase-3. Intrathecal blockage of ER stress impairs caspase-3 cleavage-dependent apoptosis in dorsal horn neurons and relieves bone cancer pain. More importantly, spinal therapy with a specific caspase-3 inhibitor Z-DEVD-FMK is enough and effective against bone cancer pain (He et al., 2019). Also, the modulation of Bax overload and caspase-3 cleavage in mitochondrial fission and apoptosis in bone cancer pain is confirmed by other research teams (Li MY. et al., 2019; Chen P. et al., 2021). Overall, this suggests that preventing ER stress-induced cellular dysfunction and caspase-3-dependent neuronal apoptosis may be a new approach for treating cancer pain (Table 2).

## Caspases and Musculoskeletal Pain

### Caspase-3

Musculoskeletal pain refers to pain in the muscles, bones, ligaments, tendons, and nerves. Chronic musculoskeletal pain patients in general show signs of peripheral/central sensitization. Dynamic recruitment of GluA1-containing AMPAR at spinal synapses contributes to central sensitization underlying pain-associated syndromes (Luo et al., 2014; Zhang et al., 2018; Wang Z. et al., 2020; Liu et al., 2020). Leucine-rich repeat transmembrane protein 1 (LRRTM1) is demonstrated to mediate post-synaptic translocation of AMPA receptor and synaptogenesis (Bhouri et al., 2018; Schroeder et al., 2018). But the regulation of LRRTM1 in pain development remains underestimated. We recently revealed that tibial fracture with intramedullary pinning causes long-lasting mechanical allodynia and cold allodynia after orthopedic surgery in mice, along with the upregulated caspase-3 activity (but not caspase-3 protein expression) and LRRTM1 expression in spinal dorsal horn (Zhang et al., 2020). Pharmacological intervention with caspase-3 specific inhibitor Z-DEVD-FMK reduces fracture-associated behavioral pain and LRRTM1-mediated alterations in excitatory synaptic plasticity. Spinal exposure to recombinant caspase-3 evoked acute pain phenotypes and spinal LRRTM1 over-expression is reversed by LRRTM1 deficiency (Zhang et al., 2020). Collectively, this demonstrates the tight interaction between caspase-3 and LRRTM1 in inducing AMPA receptor trafficking and chronic central sensitization, ultimately regulating the formation and maintenance of fracture-associated pain. Sure, it will be of great interest to investigate how fracture trauma regulates caspase-3 activation and thus mediates the onset of chronic pain (Table 2).

### Caspase-6

Our latest study provides several lines of evidence to confirm the requirement of caspase-6 in musculoskeletal pain induced by tibial fracture with intramedullary pinning (Cui W. et al., 2021). First, behavioral tests showed that tibial fractures after orthopedic operation initiate and persist postsurgical mechanical allodynia and cold allodynia, which is detectable on 3 days, peaks on 7–14 days, and sustains for at least 21 days. Second, biochemical tests found that tibial fracture up-regulates spinal active caspase-6 activity, GluA1-containing AMPA receptor trafficking, spine density in dorsal horn neurons. Third, spinal delivery of specific caspase-6 inhibitor Z-VEID-FMK and caspase-6 neutralizing antibody is sufficient to reduce the recruitment of GluA1-containing AMPA receptor at synapses and the amounts of mushroom spines, thereby attenuating fracture-associated chronic pain. Fourth, electrophysiological tests manifested that pharmacological inhibition of caspase-6 blocks AMPA receptor-mediated excitatory post-synaptic currents in the dorsal horn neurons in fracture animals (Cui W. et al., 2021). These above-mentioned results demonstrate that caspase-6-mediated changes in excitatory synaptic structure and functional plasticity is an important mechanism for the formation and maintenance of spinal nociception sensitization after fracture and orthopedic surgery, which provides a promising approach for chronic fracture pain therapy. However, there are several outstanding questions regarding how caspase-6 mediates AMPA receptor post-synaptic trafficking and ultimately triggers musculoskeletal pain. Simultaneously, in addition to the mechanism of affecting receptor transport, future researches are warranted to explore whether there are epigenetic regulations that interfere with the expression of glutamate receptors.

## Caspases and Postoperative Pain

The requirement of caspase-1 for postoperative pain development has also been clarified. Extension of laparotomy-associated postoperative pain after morphine treatment is attributed to up-regulation of inflammatory genes, including those encoding caspase-1, NLRP3, TLR4, NF-κB, IL-1β, and TNF-α (Grace et al., 2019). Thoracotomy induces persistent postoperative behavioral pain, along with the spinal up-modulation of caspase-1 and TLR4 co-localization in dorsal horn neurons (Hu et al., 2020). The decrease of mechanical withdrawal threshold is attributed to caspase-1-dependent microglial activation and the overload of inflammatory mediators (TNF-α, IL-6, and IL-1β) in spinal dorsal horn. Additionally, the alleviation of postoperative mechanical allodynia by intrathecal therapy of caspase-1 inhibitor (Ac-YVAD-CMK) is reversed after TLR4 agonist treatment (Hu et al., 2020), further indicating the tight interaction between caspase-1 and TLR4 in spinal nociception transduction and central sensitization (Table 1).

## Caspases and Opioid-Induced Hyperalgesia

**FIGURE 1:**
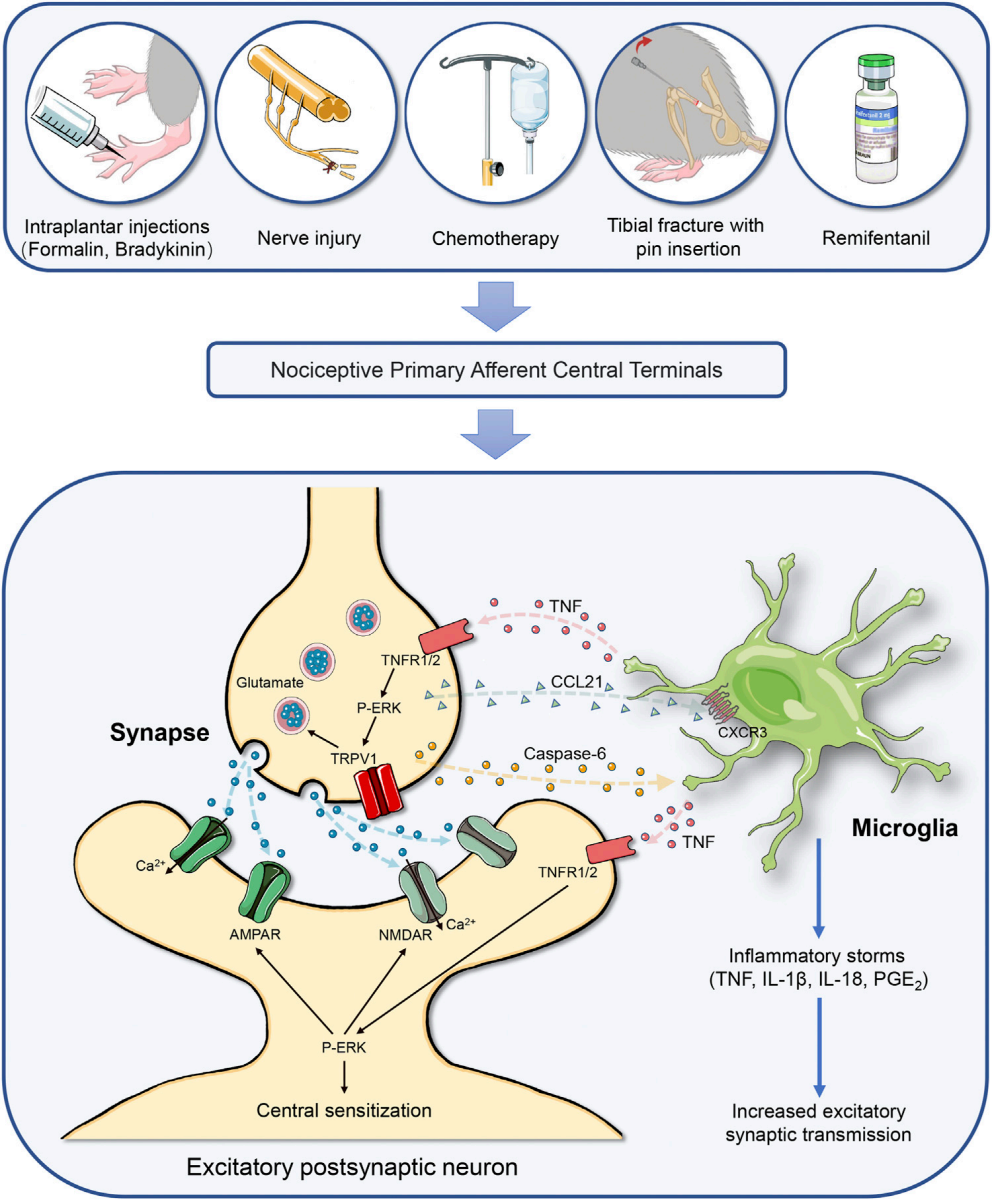
Caspase-6 cascades-mediated neuron-microglia interaction in the spinal cord initiates chronic pain through neuroinflammation and central nociception sensitization. Chronic pain is categorized as inflammatory pain, neuropathic pain, cancer pain, musculoskeletal pain, and drug treatment-induced pain, which is driven by neuroinflammation in the spinal cord dorsal horn. Painful insults, which includes peripheral tissue inflammation, nerve trauma, fracture with orthopedic surgery, chemotherapy and opioids treatment, result in the hyperexcitability of primary sensory neurons and trigger the release of caspase-6 from the central terminals of primary nociceptive afferents, which causing microgliosis and microglia activation, and subsequent microglial TNF-α secretion. Then, the interaction between TNF-α and TNFR at presynaptic sites causes the release of glutamate *via* ERK and TRPV1 pathway. Activation of TNFR at postsynaptic neurons also facilitates the phosphorylation of ERK, which drives central sensitization via positive modulations of NMDAR and AMPAR and subsequent intracellular calcium influx. Simultaneously, caspase-6 cleavage promotes chemokine CCL21 release from presynaptic neurons, which elicits microglia activation via acting on its specific receptor CXCR3. Microglia activation further increases the secretion of the pro-inflammatory mediators (IL-1β, IL-18, and PGE2). These regulations of excitatory synaptic transmission by microglial mediators at pre-synaptic, post-synaptic, and extra-synaptic sites drive central sensitization in the nociception circuits, leading to the development of chronic pain. Abbreviations: AMPAR, a-amino-3-hydroxy-5-methyl-4-isoxazolepropionic acid receptor; CCL21, chemokine (C-C motif) ligand 21; CXCR3, chemokine (C-X-C motif) receptor 3; ERK, extracellular signal-regulated kinase; IL-1β, interleukin-1β; IL-18, interleukin-18; NMDAR, N-methyl-D-aspartate receptor; p-ERK, ERK phosphorylation; PGE2, prostaglandin E2; TNF-α, tumor necrosis factor-α; TNFR, tumor necrosis factor receptor; TRPV1: transient receptor potential vanilloid-1.

Remifentanil is a potent short-acting µ-opioid agonist, regarded as an important component of balanced anesthesia in the clinical setting. Unfortunately, the intraoperative exposure to remifentanil elicits behavioral OIH phenotype in animals and patients (Zhang et al., 2017; Zhang et al., 2018; Li et al., 2021). Population-based studies also found that remifentanil can elevate peripheral mechanical nociceptive sensitivity, elicit hyperalgesia area around the wound incision, and trigger OIH, even leading to chronicity of postoperative pain (Fletcher and Martinez, 2014; Zhang et al., 2016). Chemokines and their receptors-associated neuroinflammation is essential for OIH generation (Yang et al., 2016; Zhu et al., 2017). Wang et al. (2020a) confirmed that remifentanil infusion causes mechanical allodynia and thermal hyperalgesia, along with the spinal increase in the cleavage of caspase-6, the release of CCL21 in neurons and the expression of CXCR3 in microglia. Spinal inhibition of caspase-6 activation ameliorates OIH behavior and spinal CCL21/CXCR3 accumulation. Exogenous caspase-6 also evokes acute mechanical pain and represents microglial activation, which is impaired after spinal CCL21 neutralization. This suggests the contribution of caspase-6 in CCL21 signaling in chronic pain perception. However, the downstream target molecules through which caspase-6 upregulates the synthesis of CCL21 need to be further investigated. As a result, interactions of microglia-neurons are triggered by caspase-6 activation in synaptic plasticity and the formation and maintenance of chronic pain, as the potential pain circuits in the spinal cord dorsal horn is shown in Figure 1.

## Specific Caspases Inhibitors as a Potential Candidate for Chronic Pain Treatment

Despite decades of clinical investigation and medical advancement, current approaches for chronic pain-relief are still limited (Tracey et al., 2019). Non-steroidal anti-inflammatory drugs and acetaminophen must be cautiously administered in patients with gastrointestinal diseases, renal dysfunction and hepatic insufficiency (Bindu et al., 2020; Ishitsuka et al., 2020). Tricyclic antidepressants, norepinephrine reuptake inhibitors, NMDA receptor antagonists and α_2_-δ anticonvulsants are only partially beneficial to neuropathic pain and several dose-limiting adverse-effects including sedation, somnolence and dizziness may block their practical utilization (Staahl et al., 2009; Thompson et al., 2019). Opioids, as 1st-line analgesics, frequently cause constipation, nausea, addiction, tolerance and hyperalgesia (Zhang et al., 2016; Imam et al., 2018; Colvin et al., 2019). Thus, alternative agents for pain control are urgently required. In view of the pivotal role of caspases in the pathogenesis of chronic pain, their targeting agents in the management of chronic pain have been identified as above-mentioned. The summarization and detail on therapeutic value of these drugs are shown in Table 4.

**TABLE 4:** The inhibitors targeting caspases cascades in chronic pain treatment in rodents.

Target	Inhibitors	Administration route	Rodent models of pain	References
Caspase-1	Z-YVAD-FMK	Intrathecal injection	CFA, C67BL/6 mice	Matsuoka et al. (2019)
		Intrathecal injection	LPS, Wistar rats	Clark et al. (2006)
	Ac-YVAD-CMK	Intrathecal injection	Thoracotomy, SD rats	Hu et al. (2020)
		Intraplantar injection	Hind-paw incision, C57BL/6 mice	Liang et al. (2010)
	VRTXSD727	Oral gavage	Hind-paw incision, C57BL/6 mice	Liang et al. (2010)
	VX-765	Intraperitoneal injection	SNL, SD rats	Wang et al. (2021b)
Caspase-3	Z-DEVD-FMK	Intrathecal injection	CCI, SD rats	Wu et al. (2012)
		Intrathecal injection	Walker 256 cell intraperitoneal injection, SD rats	He et al. (2019)
		Intrathecal injection	Tibial fracture, C57BL/6 mice	Zhang et al. (2020)
Caspase-6	Z-VEID-FMK	Intrathecal injection	Formalin, C57BL/6 mice	Berta et al. (2014)
		Intrathecal injection	Formalin, CD1 mice	Chen et al. (2018a)
		Intrathecal injection	SNI, SD rats	Berta et al. (2017b)
		Intrathecal injection	Tibial fracture, C57BL/6 mice	Cui et al. (2021b)
		Intrathecal injection	Remifentanil infusion, SD rats	Wang et al. (2020a)

Abbreviations: CCI, chronic constriction injury; CFA, complete Freund’s adjuvant; CPN common peroneal nerve; LPS, lipopolysaccharide; SD rats, Sprague Dawley rat; SNI, spared nerve injury; SNL, sciatic nerve ligation.

## Summary

We have summarized the role of the caspases in the development of chronic pain with different etiologies, with a view to providing new ideas for the management of chronic pain. Although significant progress has been made in preclinical study, most of the studies have only been conducted at the behavioral level, and the upstream and downstream molecular mechanisms have been poorly investigated, so there are plenty of issues that still need to be addressed. Future research should include the following directions: 1) high selective inhibitors developed for caspase-1, caspase-3 or caspase-6 can release chronic pain by inhibiting neuroinflammation, altered excitatory synaptic plasticity or neuronal apoptosis in animal models; however, whether these inhibitors can alleviate chronic pain in patients and be safely utilized in clinics remains to be explored. 2) In addition to caspases-1, 3, 6, 7, 9, and 11, it has not been conclusively established whether other members of the caspase family are also involved in the regulation of chronic pain. 3) most studies on the role of caspases in the occurrence and development of chronic pain were conducted in male animals. Considering the sex differences in the formation mechanism of chronic pain (Chen et al., 2018; Luo et al., 2018; Luo et al., 2019a; Luo et al., 2019b; Luo et al., 2021), future research should emphasize whether the caspase signaling is involved in the formation of chronic pain in female animals. 4) Despite recent advances in pain therapy, visceral pain remains poorly understood. Recent study confirms that antibiotic-induced microbial changes resulted in neuro-immune responses and visceral pain attenuation in wild type but not in caspase-1/11 knockout mice (Aguilera et al., 2021), supported the notion of the inflammasome as a promising therapeutic target in the visceral pain. Therefore, further study of caspase family and visceral pain will be a promising field. 5) It is unclear whether there is any interaction between different caspases in specific pain conditions, which needs further investigations. Anyway, the findings of the above will further provide new interventional targets for the management of chronic pain and promote the development of novel drugs related to the caspase cascades.

## Glossary

ALCAR: Acetyl-l-carnitine
AMPAR: alpha-amino-3-hydroxy-5-methyl-4-isoxazolepropionic acid receptor
ACC: anterior cingulate cortex
AD: Alzheimer’s disease
ASC: apoptosis-associated speck-like protein containing a Caspase activation and recruitment domain
ATF3: activating transcription factor-3
BAX: B-cell lymphoma 2-associated X apoptosis regulator
BDNF: brain-derived neuro-trophic factor
CCI: chronic constriction injury
CCL21: C-C Motif Chemokine Ligand 21
CFA: complete Freund’s adjuvant
CGRP: calcitonin-gene-related peptide
CIBP: cancer-induced bone pain
CIPN: chemotherapy induced neuropathic pain
COX-2: cyclooxygenase-2
CPN: common peroneal nerve
CPTP: chronic post-thoracotomy pain
CSF-CN: Cerebrospinal fluid-contacting nucleus
CXCL1: Chemokine (C-X-C motif) ligand 1
CXCR3: C-X-C Motif Chemokine Receptor 3
DAMP: damage associated molecular patterns
DRG: dorsal root ganglion
ER: endoplasmic reticulum
ERK: extracellular signal-regulated kinase
GAP-43: growth associated protein 43
GFAP: Glial fibrillary acidic protein
GSH: glutathione
G-CSF: granulocyte-colony stimulating factor
IL-1β: interleukin-1beta
LPS: lipopolysaccharide
LRRTM1: leucine-rich repeat transmembrane protein 1
LTD: long-term depression
MDA: malondialdehyde
MIP-1α: macrophage inflammatory protein-1α
mGluR1: metabotropic glutamate receptor 1
NF-κB: Nuclear Factor-κB
NLRP3: NOD-like receptor protein 3
NMDA: N-methyl-d-aspartate
NLRC4: NOD-like receptor C4
NP: neuropathic pain
3-NT: 3-nitrotyrosine
OIH: opioid-induced hyperalgesia
PD: Parkinson’s disease
p-ERK: ERK phosphorylation
PGE2: Prostaglandin E2
p-IRE1: phosphorylated inositol-requiring enzyme-1
p-PERK: phosphorylated protein kinase RNA-like endoplasmic reticulum kinase
pSNL: partial sciatic nerve ligation
RTKs: receptor tyrosine kinases
SCI: spinal cord injury model
SNI: spared nerve injury
SNL: sciatic nerve ligation
STZ: streptozotocin
TLR4: toll-like receptor 4
TNF-α: Tumor necrosis factor-α
TRPV1: transient receptor potential vanilloid-1.
